# Hereditary Transmission of Insanity

**Published:** 1845-09

**Authors:** 


*Hereditary Transmission of Insanity.*—M. Baillarger, in a recent number of the *Lancet*, gives the following result of his researches on the hereditary transmission of insanity, as to the number of cases which appear in the same family.

In six hundred families in which insanity had declared itself hereditarily, the number of lunatics at the same time was 1,466, which gives between two and three lunatics for each family. Among the most remarkable facts were the following:—

1. M. T. became insane at 17; his mother, his maternal grandmother, and a maternal uncle, had been affected with insanity.

2. M. M. became insane at the age of 20; was born of a lunatic father; his paternal grandmother had been mad, and his sister had suffered an attack of mania.

3. W. insane at 46; his mother, two uncles, a maternal aunt, and a sister, had been afflicted with insanity.

4. The father and the paternal grandfather of M. L. had been insane; he himself became a maniac at 28 years of age. Several times I have seen three brothers or sisters stricken with lunacy in the same family.

5. Madame P., aged 35, born of an insane mother, had already two brothers and a sister insane when she became maniacal.

6. In one case I found the father, the daughter, and three paternal uncles, affected with insanity.

7. In another case the mother, the daughter, and three collateral relations belonging to three different generations, a great uncle, an uncle, and a cousin were affected.—*Dublin Med. Press.*

